# Impact of Prosocial Motivation on Organizational Citizenship Behavior and Organizational Commitment: The Mediating Role of Managerial Support

**DOI:** 10.3390/ejihpe11020032

**Published:** 2021-05-21

**Authors:** Muhammad Arshad, Ghulam Abid, Francoise Contreras, Natasha Saman Elahi, Muhammad Ahsan Athar

**Affiliations:** 1 School of Business Administration, National College of Business Administration & Economics, Lahore 54660, Pakistan; natashasaman20@gmail.com (N.S.E.); ahsanchaudhry59@gmail.com (M.A.A.); 2 Department of Business Studies, Kinnaird College for Women, Lahore 54000, Pakistan; ghulam.abid@kinnaird.edu.pk; 3 Escuela de Administración, Universidad Del Rosario, Bogota 541030, Colombia; francoise.contreras@urosario.edu.co

**Keywords:** prosocial motivation, managerial support, organizational citizenship behavior, organizational commitment

## Abstract

This study, based on the conservation of resources (COR) theory, explores the impact of contextual variables, such as prosocial motivation, on employee discretionary behavior and organizational commitment. The mediating mechanism of managerial support at work defines the nature of the proposed relationships. Data from 303 administrative, instructional, and supervisory staff—predominantly male (95%) and with an average age of 30 years—working on Technical and Vocational Education and Training (TVET) for Pakistan’s public sector were collected and analyzed by employing SPSS version 24. Confirmatory factor analyses suggested a good fit model, while a correlation matrix provided a significant and positive effect of prosocial motivation on employee citizenship behaviour and organizational commitment. Managerial support mediated the relationship between prosocial motivation and the employees’ organizational commitment and citizenship behaviour. The theoretical and practical implications discussed in this study seek to guide the management area to promote managerial support for better outcomes. These outcomes have considerable tactical, statistical, and real-world inferences for the stakeholders of the TVET sector.

## 1. Introduction

Due to its significant contribution to sustainable country development, the technical and Vocational Education and Training sector has become a driving force and is drawing global attention [1]. In developing countries where the workforce is enormous, the TVET sector plays a vital role by developing human resources to promote the structure of economic development [2]. Pakistan, being an emerging economic force, understands the importance of human resources development engaged in promoting the TVET sector in the country [3]. Prosocial employees are valuable resources for the country’s development [4] from the perspective of the TVET sector, where thousands of trainees are receiving technical and vocational skills. Previous research suggests that prosocial motivation, which refers to the desire to help others [5], has a positive impact on a number of work outcomes, such as the employee’s connection with the workplace [6,7], citizenship behavior [4,8], and organizational performance [9]. However, without managerial support, prosocial employees may not necessarily produce sustainable outcomes, and the achieved success may only be temporary and disappear over time. Thus, it is important to inspect the boundaries of the conditions under which prosocially motivated employees may develop a working environment that is conducive to achieving the organizational objectives. This study is then based on the assumptions of the conservation of resources theory, which investigates the indirect influence of prosocial motivation on organizational commitment and discretionary behavior through managerial support. The COR theory argues that individuals are motivated to protect, procure, and preserve the resources of an organization when they perceive organizational support [10]. These relationships of social exchange evolve when employers appreciate the employees’ stance, thus entailing positive consequences in the long run [11]. Previous literature reviews suggest that despite the importance of this topic, very few studies have explored the contextual factors that may promote the positive effects of prosocial motivation. They also recommend that employees’ prosocial motivation exert positive influence on their citizenship behavior in the conducive working environments, duly supported by the supervisors [7]. This study observes the indirect impact of prosocial motivation on OCB and organizational commitment [OC] through the mediating mechanism of managerial support in the context of the TVET in the public sector.

Managerial Support is depicted as the perception by employees that their supervisors provide them with support towards their new and innovative ideas [12]. This support is an integral part in the smooth execution of organizational objectives [13], particularly in the TVET sector, where thousands of trainees of lower strata of society are engaged in acquiring technical and vocational skills [14]. Similarly, managerial support is important in creating an influence on the overall organizational behavior of the employees [15]. Our study postulates that managerial support can also enhance the employees’ discretionary behavior which may be presumed to be a kind of prosocial or voluntary behavior considered by organizational fellows and, in turn, provide social settings that support the achievement of organizational goals while improving task performance [16]. This employee behavior thus plays a significant role in improving the job performance and achieving the organizational desired goals [17].

Organizational commitment refers to the employees’ emotional attachment to the organization [18,19]. Previous studies have explored positive effects of employee prosocial behavior [20], self-efficacy [21], and thriving at work [22] on organizational commitment [20]. However, the contextual factors relating to the circumstances where prosocial motivation fosters organizational commitment remain less explored. Therefore, this study empirically examines the role of managerial support in the relationship between employees’ prosocial motivation and organizational commitment. Bearing the former claims in mind, organizational commitment is a highly researched job attitude in the field of organizational behavior, management [19,23], human resources management [24], and organizational psychology [25]. This issue has attracted extensive attention from scholars and practitioners due to its positive organizational and individual outcomes such as higher productivity, job performance [26], better work-life balance, workplace stability, employee satisfaction [27,28], organizational citizenship behavior, better attendance, employee retention, and lower turnover intention [29], among others. The relationship between managerial support and employee commitment is less explored in the Asian context. In this regard, we desire to provide evidence that managerial support could be an important contributor to organizational commitment.

Extending the discussion further, managerial support at the organizational level is presumed to promote the employees’ citizenship behavior. Organizational citizenship behavior is the proactive discretionary behavior to voluntarily contribute to the organization [30] displayed by an individual. Previous research has explored the positive impact of prosocial motivation on employee discretionary behavior [4]. This study, however, is interested in investigating the impact of managerial support on the relationship between the employees’ prosocial motivation and their citizenship behavior in the context of the TVET sector.

Furthermore, this research has empirically examined the influence of prosocial motivation on managerial support. Prosocial motivation is a motivational power that appreciates the efforts of employees [31]. According to researcher [5], prosocial motivation is a desire to exert effort to value other people. This behavior carries a significant impact on employees’ working in the communal sector, particularly those of the public TVET sector, are meant to serve the common people. We are also interested in examining the indirect effect of the prosocial motivation of employees serving in the public sector with organizational commitment and organizational citizenship behavior with the help of managerial support. The past literature explores the relationship between these and other variables, but there are limited studies that explore the impact of prosocial motivation on organizational citizenship behavior and organizational commitment in the public sector. Thus, the present research seeks to answer the following research questions in the public sector TVET organizations in Pakistan:

Is there any connection between prosocial motivation and managerial support?

Does managerial support affect organizational commitment and organizational citizenship behavior?

Does managerial support mediate the relationship between prosocial motivation and organizational commitment as well as prosocial motivation and organizational citizenship behavior?

## 2. Theoretical Perspective

This research is supported by the conservation of resources theory proposed initially by [32], which has been applied in studies of supervisor–subordinate relationships [33]. According to this theory, when employees are motivated and seek to conserve new resources, organizations obtain optimistic assistance from their workers as give-and-take for providing assistance to their workers. The more the organizational management supports its workers well, the more the workers will respond in multiplication by demonstrating organizational commitment and organizational citizenship behavior [34]. Mainly, the commitment of workers to their organization increases as a result of managerial support that advances organizational performance. In sum, the present research seeks to test the relationships between prosocial motivation and organizational citizenship behavior and organizational commitment, exploring the mediating role of managerial support in the assumptions of conservation of resources theory. This discussion promotes the notion that prosocially motivated employees serving in the public TVET sector, would procure and preserve the resources of educational institutes engaged in disseminating technical education and vocational skills to the workforce of economically downtrodden classes in developing countries like Pakistan.

### 2.1. Prosocial Motivation and Managerial Support

The coordination and cooperation between employees and management may ease the execution of challenging tasks; therefore, employees’ prosocial behaviour always serves as a contributing factor in this perspective [35]. The desire to help colleagues promotes the welfare of others and also frames the notion that people are the most valuable asset for any organization [36]. Supported in studies [37,38] asserts that motivated people are those who are primarily concerned about their contribution to the benefit of others, ignoring a personal return. These are employees who achieve real long-term success, dealing in a better way with the organizational stressors. Prosocially motivated employees are not usually discouraged by daily troubles because they find their work valuable and meaningful [39]. Due to their willingness to contribute, these employees are the preferred choice for the organizations obtaining support from supervisors and managers [35]. This kind of behavior works in different dimensions, benefiting the organization and promoting the proactive behavior that finally gets administrative support [40]. In light of the above discussion, we derive the following hypothesis:

**Hypothesis** **1** **(H1).** *Prosocial motivation is positively associated with managerial support.*

### 2.2. Managerial Support and Organizational Citizenship Behavior

Employees demonstrating high discretionary behaviour support their supervisors, managers and colleagues in the high times and always remain concerned about their partners’ well-being [41]. Normally, these employees show an ethical behavior in the workplace [42] and go beyond the expectations of job demands. Similarly, organizational citizenship behavior has been associated with positive organizational outcomes and employee performance [43]. Employee participation in the organizational affairs in the work context demonstrates their contentment and support backed by supervisors and managers [44]. Furthermore, previous research has discussed that cordial relationships between employees and management areas keep employees motivated to pursue their assigned tasks within allocated times and resources [45]. They, based on the assumption of the COR theory, invest their potential and abilities in the promotion of organizational objectives.

The appreciation of organizational support promotes more positive relationships among workers and their managers and instigates organizational citizenship behavior in junior employees [46]. Employees with an optimistic attitude appreciate management support, which signifies an attachment to the organization that mitigates turnover intention [47]. Consequently, their discretionary behaviour may be strengthened. We assume that, in the public sector, the demonstration of employee discretionary behavior is an expression of support from their colleagues and management that motivates employees to extend their cooperation to their supervisors and the dealings of the workplace. This also supports the notion that employees who demonstrate citizenship behavior are actively involved in solving citizens’ issues, vigorously recognizing problems concerning the current public service. They also advise the best possible solutions to their communities. This discussion supports the notion that managerial support leads to a positive impact on employees’ OCB, thus we propose the following hypothesis:

**Hypothesis** **2** **(H2).** *Managerial support is positively associated with employee discretionary behavior.*

### 2.3. Managerial Support and Organizational Commitment

Managerial support at an organizational level is a vital factor for workers to build trust [48] and achieve organizational goals [49]. Organizations expect supervisors or leaders to play a leading role by pursing employee’s motivation. On the other hand, employees pursue managers who assess their performance reports as agents of the organization. Consequently, workers receive support from their supervisors as organizational support [45]. Employees who perceive their supervisors as competent, trustworthy, and supportable are more motivated to share the organizational goals by demonstrating their OC. To achieve this high standard of organizational commitment from employees, supervisors with a democratic leadership style allow employees to participate in the decision-making procedure, which promotes their confidence and, resultantly, increases their performance [50]. Thus, managerial support may improve or decrease the OC of employees. Research has found evidence about the positive association of managerial support and organizational commitment, i.e., it was found that high levels of organizational assistance are related to a high organizational commitment from employees [51,52]. On the contrary, the lack or scarcity of managerial support of employees decreases their organizational commitment and causes poor performance, also encouraging turnover, absenteeism, and stress [53]. So, on the basis of the above discussion, we propose the following hypothesis:

**Hypothesis** **3** **(H3).** *Managerial support is positively associated with organizational commitment.*

### 2.4. Managerial Support: Prosocial Motivation and Organizational Citizenship Behavior

Research has already explored the association between the prosocial motivation of individuals and supportive behaviors by management [54]. Another study by scholars [55] has dignified an association between two prosocial personality features and volunteerism, and found that prosocial personality features have important links to a commitment to volunteer deeds. Management of the organization always encourages and supports the prosocially motivated employees. The impact of the multiplication of the support of management and prosocial motivation spurs the organizational citizenship behavior of the employees. This notion is hypothesized as:

**Hypothesis** **4** **(H4).** *Managerial support mediates the association between prosocial motivation and organizational citizenship behaviors.*

### 2.5. Managerial Support: Prosocial Motivation and Organizational Commitment

Organizations expect commitment from workers in order to decrease expenses and improve performance and provision of product quality [56]. This commitment can be improved by building beliefs, cooperation, and employee empowerment. These approaches were often established in the non-profit sector but are now being progressively and effectually employed by the profit sector as well. Organizations should spend on HR and retain employees in order to achieve organizational objectives. The higher-level organizational pledge and prosocial motivation are associated with managerial support and the managerial working style [50]. Similarly, motivated employees within a supportive environment encourage managers to involve them in the decision-making processes which, in turn, increase the employees’ commitment to achieve the objectives of the organization. Thus, we posit that managerial support may mediate the relationship between prosocial motivation and the OC of workers.

**Hypothesis** **5** **(H5).** *Managerial support mediates the relationship between prosocial motivation and organizational commitment.*

### 2.6. Research Model

**Figure 1:**
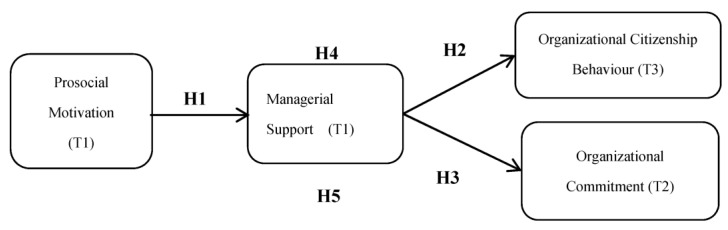
Conceptual Framework.

The literature suggests that there is a positive relationship between prosocial motivation and managerial support. Similarly, managerial support is positively associated with OCB. Managerial support may be positively associated with OC. The literature also suggests that managerial support mediates the relationship between prosocial motivation and OCB. Managerial support can also mediate the proposed relationship between prosocial motivation and OC. The conceptual model of this study, outlined in light of the review of literature is shown in Figure 1.

## 3. Method

### 3.1. Participants and Procedure

The study was carried out in the Technical and Vocational Education Training [TVET] sector of Pakistan. The targeted respondents belonged to two categories: managerial and instructional staff. In the first category, the respondents were managerial and administrative staff working at the head and at a field office of technical education in Punjab, the largest province of Pakistan. The staff was comprised of strategic and operational management including the general managers, deputy general managers, managers/directors, deputy managers, assistant managers, and their supporting staff. In the second category, the respondents were instructional staff, which included principals, heads of departments, instructors, and support staff of the different short courses/technologies engaged at the Govt. College of Technologies and Govt. Technical Training Institutes under the administrative control of the Punjab Technical Educational and Vocational Training Authority (PTEVTA]. PTEVTA supervises more than 400 technical and vocational training institutes, where about 0.2 million trainees are enrolled annually in different trades and technologies [14].

A self-administered questionnaire was used for the data collection process. This study was cross-sectional and counted with a time-lagged structure as the data were collected at a three-wave period with a two-week pause between each wave, used as a multi-source for the removal of common method biases. As suggested by [57], in this study data was collected from employees and from their supervisors. Furthermore, to observe the possible change in the predictor variables, a temporal three-wave design was adopted to avoid common method biases. A team comprised of five members was trained and guided to apply the questionnaires, and a cover letter was sent beforehand to the heads of the sections and departments. This letter communicated the goal of this research, namely, to examine the attitudes of staff pertaining to prosocial motivation, managerial support, OC, and OCB. Participants were encouraged to respond as accurately as possible and were assured that their participation would be kept confidential and anonymous. Furthermore, they were told that the data would be used for academic research purposes only.

A total of 370 questionnaires were distributed to managerial/administrative and instructional staff at time period 1 [T1]. Out of 370 distributed questionnaires, 350 completed questionnaires [T1] were received. At time period 2 [T2], a total of 350 questionnaires were distributed again to those respondents who responded at time 1 and 335 completed questionnaires were received back. At time 3 [T3], we contacted the respondents’ immediate boss to gather data regarding the organizational citizenship behavior towards the organization. A total of 30 bosses/supervisors responded 303 questionnaires. Participants were predominantly male [95%] with an average age of 30 years and more than half of the participants belonged to supporting staff. Participants had been recruited 5 to 20 years before and had stayed in their current ranks/positions for a range varying from of almost 11 months to 10 years; 32% of respondents were supporting staff. Furthermore, the education level of the supporting staff ranged from intermediate education to master’s degrees, whereas the qualification of instructional staff ranged from a diploma in associate engineering to graduate and post-graduate engineering studies. The heads of departments/sections and principals/HOIs were holders of engineering degrees, master‘s degrees in business administration, and PhDs. The average age of principals/supervisors was 42 and their qualification level varied from master/engineering to postgraduate. The span of control for each manager varied from person to person; however, the range of span of control was 5–10 personals in case of managerial offices in head and field offices.

### 3.2. Measure

All items in the questionnaire with the exception of demographic characteristics were measured on a five-point Likert type scale from five [strongly agree] to one [strongly disagree]. Prosocial motivation was measured with five items based on validated measures reported in prior research [58]. The sample items from the scale were, “I do my best when I am working on a task that contributes to the well-being of others” and “I like to work on tasks that have a potential to benefit others”. The average score of responses from employees other than the general manager/deputy general managers/principal was used to compute this measure. Cronbach’s alpha was 0.80, representing a higher level of internal reliability for the used scale.

Managerial Support was measured through six items based on validated measurements reported in prior research [59]. The sample items from the scale were “my supervisor is supportive when I have a problem at work” and “my supervisor accommodates me when I have family or personal business to take care of”, for example, medical appointments, meetings with the child’s teachers, etc. The average score of responses from employees other than the general manager/deputy general managers/principal was used to compute this measurement. The combined scale, designed by combining the values of the six items that had a Cronbach’s alpha score of 0.82, showed a higher level of internal reliability.

Organizational commitment was measured using a nine-item scale developed by [60]. This scale includes three basic components of organizational commitment: identification [three-item], involvement [three-item], and devotion [three-item]. Sample items: “I talk about this organization to my friends as a great organization I work for” [identification], “I am proud to tell others that I am part of this organization” [involvement], and “What this organization stands for is important to me” [loyalty]. In this study, the variable was taken as a composite variable. Cronbach’s alpha was 0.86, indicating a higher level of internal reliability for the used scale.

Organizational Citizenship Behavior was measured using the eight validated items developed by [61]. This scale comprises eight statements for OCB focused on individuals within the organization [OCB]. The sample items from the scale were “this employee attends functions that are not required but that help the organizational image” and “my supervisor accommodates me when this employee defends the organization against other employees criticizing it.”. The average score of responses from employees other than the general manager/deputy general managers/principal was used to compute this measure. Cronbach’s alpha was 0.85, signifying a higher level of internal reliability for the used scale.

### 3.3. Control Variables

Past studies suggest a potential relationship between demographic characteristics and both workplace organizational citizenship behavior and organizational commitment [62]. Thus, gender, age, education level, organizational tenure, job level, and marital status were carefully controlled, avoiding potential effects from demographic variables on resultant variables [OCB and OC].

## 4. Analysis and Results

CFA was applied to check the data’s factor structure following the traditions [63]. After CFA, descriptive statistics were used to understand the data. This led the researchers to use a correlation matrix, useful for understanding the relationship among the study’s variables. Finally, the regression analysis helped in verifying the hypotheses included in this study.

### 4.1. Analysis and Results

A measurement model using AMOS 24 reveals the factor structure based on the validated measurement scales that are used. Four latent factors were distinguished: prosocial motivation, managerial support, OCB, and organizational commitment. CFA specifies that the associations between all observed variables and their respective latent dimensions are statistically significant. The chi-square statistical model is divided by degrees of freedom 1.96, CFI 0.90 and a Root Mean Square Error of Approximation [RMSEA] 0.06. The modified measurement model shows that all observed variables associated to their latent constructs are statistically significant. The fit indices show an acceptable fit to the data (Table 1).

Table 2 shows the convergent and discriminant validity of our studied model. It shows a composite reliability of variables above the standard, which is 0.70. The average variance extracted [AVE] is also above the standard [0.5], which means that convergent validity of the model shows model fitness. Discriminant validity also shows model fitness. So, both results show that the model is fit and valid.

Means [M], SD, range[R], and number of observations [N] of all variables is described in Table 3. The outcome shows that the mean of prosocial motivation is fairly high, with a mean score of 4.33 on a five-point scale. Certainly, it makes sense that employees working in the public TVET sector demonstrate a high degree of prosocial motivation. The scores stated below arise from the prosocial motivation construct which is 1.40 (Table 1). In contrast to the other impressions that are measured on a five-point Likert scale, the SD of PM is also relatively small, i.e., 0.53. Another prominent result is the relatively low score on managerial support [3.87], which matches with the top-down direction in which policy reform needs to be employed. The table also explains the reliability analysis of the study variable. It shows that the Cronbach alpha reliability of prosocial motivation is 0.80, managerial support [0.82], organizational commitment [0.85], and organizational citizenship behavior [0.86]. All of these are above the standard, which is 0.7.

The bivariate correlations among all variables are shown (Table 3). Correlational analysis indicates that there is a statistically significant correlation among the dependent variable, i.e., managerial support, and each concept that was identified in the CFA. The relationship of prosocial motivation with managerial support is strong [r = 0.30, *p* < 0.05]. Similarly, the associations between managerial support and organizational commitment are also particularly strong [r = 0.37, *p* < 0.05]. Prosocial motivation is not related to organizational citizenship behavior [r = 0.06, *p* > 0.05]. Correlation of management support is also significantly strong with organizational citizenship behavior [r = 0.15, *p* < 0.05]. The employees of the public TVET sector organization in Pakistan consider management support for their OCB and organizational commitment. The correlation matrix further shows a significant relation between OCB and organizational commitment [r = 0.13, *p* < 0.05].

### 4.2. Hypotheses Testing

To verify the research’s hypotheses, a PROCESS Macros [Model 4] was used [Hayes, 2013]. Two separate models are projected. Model 1 includes prosocial motivation and managerial support. It is observed that there is a significant correlation between prosocial motivation and managerial support [B = 0.36, *p* < 0.05], which supports hypothesis H1 as shown in Table 3. Similarly, Model 2 shows a significant relationship between managerial support and organizational citizenship behavior [B = 0.12, *p* < 0.05]. Similarly, in Model 4, there is a significant relationship between managerial support and organizational commitment [B = 0.32, *p* < 0.05]. In Model 3, the table shows that prosocial motivation is not significantly related to organization citizenship behavior [B = 0.02, *p* > 0.05]. Therefore, H2 of the study is not supported by the data. Model 5 shows that prosocial motivation is not positively related to organizational commitment [B = 0.15, *p* > 0.05], therefore H3 of the study is not supported by data either (Table 4).

Furthermore, there is no direct significant relationship between prosocial motivation and the dependent variable, namely organizational citizenship behavior, although the indirect impact of prosocial motivation was checked through the construct, i.e., managerial support. It was observed that managerial support mediates the significant relationship between prosocial motivation and organizational citizenship behavior [B = 0.05, *p* = 0.00], which is shown in Table 5. Here, hypothesis H4 is reported to support the relationship between prosocial motivation and organizational citizenship behavior. Although it is observed that there is no direct significant relationship between prosocial motivation and organizational commitment, the indirect impact of prosocial motivation appears through the construct (i.e., managerial support). It is also observed that managerial support mediates the significant relationship between prosocial motivation and organizational commitment [B = 0.12, *p* = 0.00] as shown in Table 5. Here, hypothesis H5 supports the relationship between prosocial motivation and organizational citizenship behavior.

## 5. Discussion

The present study aims to investigate the impact of prosocial motivation on the OCB and OC of employees, exploring the mediating role of managerial support in the public TVET sector in Pakistan. This research also highlights that the support that workers perceive from managers and the organization leads employees to demonstrate a response in terms of OCBs and OC. It also guides management of the TVET sector to promote the prosocially motivated employees to demonstrate a better performance. According to the results, the first hypothesis is supported: prosocial motivation shows a significantly positive relationship with managerial support. These results are consistent with a previous study [64] and the findings suggest that managerial support to the motivated employees may promote the positive conduct of their employees with the masses.

The results also provide a significantly positive relationship between prosocial motivation and OCB and relationship is mediated by the managerial support. Associations are further supported by the regression analysis results where prosocial motivation is positively related to OCB. Thus, the fourth hypothesis was also supported. This finding corresponds with previous studies [65,66]. Outcomes also propose that the workers getting support from their management were motivated and pay back to the organization by demonstrating OCB and OC, which is significantly vital for the promotion of the TVET’s image in developing countries. Thus, workers get involved in citizenship behaviors to respond to managerial support provided by the organization. The second hypothesis is also supported: management support positively predicts the OCB. This is to say that workers who have a greater organizational commitment are more likely to perform additional role behaviors, which are very important for organizations. The fifth hypothesis is concerned with the mediating role between the relationship of prosocial motivation and OC that managerial support plays. It seems that when individuals perceive support from the organization and from their managers, they can feel energized and enthusiastic to perform better at their work.

Results provide useful information for managers working in the public TVET sector where the main objective is to facilitate and provide services to common people. Recently, ref. [54] found that employees’ commitment to their organization can produce changes in the public sector. Likewise, since this study highlights the impact of prosocial motivation behavior on OCB and OC in coordination with managerial support, the managers can reduce the distance between employees and higher managerial staff as they are in permanent interaction with both of them at the same time. Research shows that managerial support has a promising association with organizational commitment: the higher the level of organizational support that the employees feel, the higher their commitment towards the organization. Therefore, management support fosters organizational citizenship behavior.

### Theoretical and Practical Contribution

This research was carried out in the public TVET sector, where the objective of organizations is to serve the common people. Theoretically it adds new knowledge indicating that managerial support plays a vital role in improving the OC and OCB of TVET sector employees. Supportive management indirectly motivates and enhances employees’ efficiency. This research also has imperative practical implications for the managers of the TVET sector, where TVET institutions are engaged in serving the poor sectors of the society. If higher management of the TVET sector were to support the operational management, this would enhance their OC and OCB. The highly motivated operational management would perform their duties more enthusiastically and the lower strata of society, whose children are receiving technical and vocational training, would realize their impact. Furthermore, this skilled workforce would be an important source for contribution to the feeble economy of Pakistan, which can be applied to other developing countries.

## 6. Limitations and Future Directions

This research carries some limitations. First, the test was focused only on a single public TVET sector organization, thus its findings cannot be generalized to other sectors and organizations in Pakistan. Therefore, we suggest replicating this model in other sectors in order to generalize the findings in other cities of Pakistan and in other developing countries. These limitations are in line with the suggestion of [67], which suggests the collection of data from more than one organization. The study was conducted only in the public sector; however, this model may be tested on the private sector as well, and also in the other countries of the world. The second limitation of this study is that its findings are based on the data collected from 303 respondents, therefore, the results may not be applicable to the whole TVET sector. Third, this study was carried out in Pakistan, which is a developing country; therefore, results may vary if study is conducted in developed countries, where working environment and HR practices are observed differently. Future research may include collecting data from a larger population in order for the results to be more appropriate and meaningful. We also encourage future researchers to consider other mediators such as family support in these relationships. Further studies may include some strong moderator, which is useful to the study settings. However, apart from the limitations, this study highlights important findings regarding the impact of prosocial motivation on employee OCB, and the mediating role of managerial support between the relationship of prosocial motivation and OCB.

## Figures and Tables

**Table 1:** Confirmatory Factor Analysis.

	CFI	GFI	RMR	TLI	NFI	RMSEA
Four factor Model	0.90	0.92	0.91	0.91	0.93	0.06
Three factor Model	0.85	0.82	0.86	0.84	0.79	0.09
Two factor Model	0.74	0.78	0.82	0.72	0.69	0.12
One factor Model	0.73	0.72	0.69	0.71	0.68	0.13

Note: Four Factor Model [“Prosocial Motivation”, “Managerial Support”, “Organizational Citizenship Behavior”, “Organizational Commitment”]. Three Factor Model [“Prosocial Motivation”, “Managerial Support”, “Organizational Citizenship Behavior, Organizational Commitment”]. Two Factor Model [“Prosocial Motivation, Managerial Support”, “Organizational Citizenship Behavior, Organizational Commitment”]. One Factor Model [“Prosocial Motivation, Managerial Support, Organizational Citizenship Behavior, Organizational Commitment”].

**Table 2:** Convergent and Discriminant Validity.

	CR	AVE	MSV	ASV	PM	MS	OCB	OC
PM	0.80	0.62	0.35	0.20	0.66			
MS	0.82	0.59	0.25	0.12	0.49	0.79		
OCB	0.85	0.62	0.63	0.28	0.36	0.50	0.77	
OC	0.86	0.71	0.54	0.25	0.21	0.23	0.31	0.68

**Table 3:** Descriptive Statistics and Correlations Matrix.

Variables	Mean	S.D	1	2	3	4	5	6	7	8	9
1.Martial Status	1.76	0.42	1								
2. Age	3.11	0.32	0.54 **	1							
3. Designation	1.78	0.24	0.03	0.09	1						
4. Experience	2.65	0.51	0.47 **	0.86 **	0.07	1					
5.Qualification	3.44	0.58	−0.12 *	−0.30 **	0.05	0.32 **	1				
6. Prosocial Motivation	4.33	0.53	0.10	0.09	−0.02	0.10	0.05	**0.80**			
7. Managerial Support	3.87	0.67	0.01	0.11	−0.06	0.11	−0.03	0.30 **	**0.82**		
8.Organizational Commitment	3.87	0.59	0.01	0.06	0	0.06	−0.06	0.14 *	0.37 **	**0.85**	
9.Organizational Citizenship Behavior	4.00	0.57	−0.04	−0.07	−0.08	−0.04	0.05	0.06	0.15 **	0.13 *	**0.86**

Note: ** Correlation is significant at the 0.01 level(2-tailed). * Correlation is significant at the 0.05 level(2-tailed). Cronbach’s alphas are on the diagonal (highlighted).

**Table 4:** Results of Regression Analysis.

Variables	Managerial Support	Organization Citizenship Behavior		Organizational Commitment	
	Model 1	Model 2	Model 3	Model 4	Model 5
Independent					
Prosocial Motivation	0.36 **		0.02		0.15
Mediator					
Managerial Support		0.12 **		0.32 **	

Note: ** Correlation is significant at the 0.01 level(2-tailed).

**Table 5:** Results of Mediation Analysis.

Mediation	β	Sig.	LLCI	ULCI
PM 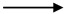 MS 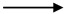 OCB	0.05	0.00	0.02	0.16
PM 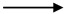 MS 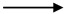 OC	0.12	0.00	0.07	0.18

Note: PM: Prosocial motivation, MS: Managerial support, OCB: Organizational citizenship behavior, OC: Organizational commitment.

## Data Availability

The datasets generated and analyzed in the current study are available from the corresponding author upon reasonable request.
